# Application of Supervised Machine Learning for Behavioral Biomarkers of Autism Spectrum Disorder Based on Electrodermal Activity and Virtual Reality

**DOI:** 10.3389/fnhum.2020.00090

**Published:** 2020-04-03

**Authors:** Mariano Alcañiz Raya, Irene Alice Chicchi Giglioli, Javier Marín-Morales, Juan L. Higuera-Trujillo, Elena Olmos, Maria E. Minissi, Gonzalo Teruel Garcia, Marian Sirera, Luis Abad

**Affiliations:** ^1^Instituto de Investigación e Innovación en Bioingeniería, Universitat Politécnica de Valencia, Valencia, Spain; ^2^Red Cenit, Centros de Desarrollo Cognitivo, Valencia, Spain

**Keywords:** autism spectrum disorder, sensory dysfunction, virtual reality, electrodermal activity, assessment

## Abstract

**Objective:**

Sensory processing is the ability to capture, elaborate, and integrate information through the five senses and is impaired in over 90% of children with autism spectrum disorder (ASD). The ASD population shows hyper–hypo sensitiveness to sensory stimuli that can generate alteration in information processing, affecting cognitive and social responses to daily life situations. Structured and semi-structured interviews are generally used for ASD assessment, and the evaluation relies on the examiner’s subjectivity and expertise, which can lead to misleading outcomes. Recently, there has been a growing need for more objective, reliable, and valid diagnostic measures, such as biomarkers, to distinguish typical from atypical functioning and to reliably track the progression of the illness, helping to diagnose ASD. Implicit measures and ecological valid settings have been showing high accuracy on predicting outcomes and correctly classifying populations in categories.

**Methods:**

Two experiments investigated whether sensory processing can discriminate between ASD and typical development (TD) populations using electrodermal activity (EDA) in two multimodal virtual environments (VE): forest VE and city VE. In the first experiment, 24 children with ASD diagnosis and 30 TDs participated in both virtual experiences, and changes in EDA have been recorded before and during the presentation of visual, auditive, and olfactive stimuli. In the second experiment, 40 children have been added to test the model of experiment 1.

**Results:**

The first exploratory results on EDA comparison models showed that the integration of visual, auditive, and olfactive stimuli in the forest environment provided higher accuracy (90.3%) on sensory dysfunction discrimination than specific stimuli. In the second experiment, 92 subjects experienced the forest VE, and results on 72 subjects showed that stimuli integration achieved an accuracy of 83.33%. The final confirmatory test set (*n* = 20) achieved 85% accuracy, simulating a real application of the models. Further relevant result concerns the visual stimuli condition in the first experiment, which achieved 84.6% of accuracy in recognizing ASD sensory dysfunction.

**Conclusion:**

According to our studies’ results, implicit measures, such as EDA, and ecological valid settings can represent valid quantitative methods, along with traditional assessment measures, to classify ASD population, enhancing knowledge on the development of relevant specific treatments.

## Introduction

Autism spectrum disorder (ASD) is a neurodevelopment disorder characterized by a wide range of impairments, ranging from social to physical and cognitive functions (Baron-Cohen, 1990), affecting one in 160 children (World Health Organization [WHO], 2019). ASD symptoms arise as early as 2 to 4 years in age, and in some cases, the signs of ASD might start as early as 6 months old (Lord et al., 2006; Anagnostou et al., 2014). Specifically, ASD is associated with social and interaction symptoms as well as stereotyped and repetitive behavior patterns (American Psychiatric Association, 2013) that have a significant impact on educational (Levy and Perry, 2011) and social life (Schmidt et al., 2015). Furthermore, sensory processing dysfunctions have been observed as a relevant aspect of ASD symptomatology; indeed it is experienced by over 90% of ASD children (Leekam et al., 2007; Tomchek and Dunn, 2007; Baron-Cohen et al., 2009). Sensory processing is the ability to capture, elaborate, and integrate information through the five senses (touch, movement, smell, taste, vision, and hearing), allowing adapting behavioral responses to the environment (Miller et al., 2007). In the ASD population, such sensory processing and integration of stimuli are experienced differently from that of the typical development (TD) population, affecting response to stimuli. In more details, they show hyper-sensitivities (over-responsiveness) and hypo-sensitivities (under-responsiveness) to a wide range of sensory stimuli. Previous studies on sensory dysfunctions showed a hypersensitivity to visual and auditive stimuli, such as bright lights or noisy sounds (Tomchek and Dunn, 2007; Baron-Cohen et al., 2009; Tomchek et al., 2014); conversely, with olfactive stimuli, they present hypo-sensitiveness in detecting odor threshold (Dudova et al., 2011; Ashwin et al., 2014). Sensory dysfunction consequently affects the information processing in ASD, and it has been suggested that it may be the cause of impairments in several psychological domains, such as in cognitive and social responses (Tomchek and Dunn, 2007; Baron-Cohen et al., 2009).

### Current Issues in ASD Diagnosis and the Need for Biomarkers in ASD

Traditionally, ASD diagnosis and assessment include a series of explicit qualitative and quantitative measures characterized by semi-structured behavioral tasks’ observations in which the examiner rates and scores an individual’s responses to prompted situations (e.g., the Autism Diagnostic Observation Schedule, ADOS; Lord et al., 1999) and family structured interview (e.g., the Autism Diagnostic Interview-Revised, ADI-R; Lord et al., 1994). For example, the ADOS measure consists of various standardized activities introduced by the examiner, such as a simulation of having a snack together, that permits to observe the occurrence or non-occurrence of behaviors related to ASD. ADOS principally focuses on social behavior and communication analysis, and it is characterized by five different modules that allow tailoring of assessment to the age and communication development of the participants. Regarding sensory processing, the utmost test for its evaluation is Sensory Profile-2 (Dunn, 2014), a qualitative questionnaire in which family caregivers answer to several questions about activities at home, in school, and in the community (see the section “Materials and Methods” for test description).

Despite these instruments having been widely adopted in ASD research and clinical practice, several limitations remain (Volkmar et al., 2009), mainly regarding the absence of explicit sensory functioning assessment, the subjective evaluation and the examiner’s expertise, and the ecological validity of the assessment setting.

Concerning the first limitation, traditional assessments have been designed following both ASD ICD-10 and DSM IV guidelines that do not consider sensory dysfunction as a necessary and distinct diagnostic criterion. Thus, ADI-R and ADOS do not tap sensory processing and responsiveness (Leekam et al., 2007). Second, training in administration and scoring is crucial and highly recommended (Lord et al., 2001) since test results and diagnosis rely on the examiner’s subjective ability to detect ASD-related features. Examiners who not have a high level of ASD-specific previous training and expertise might lead to inappropriate task presentation and administration. This could influence the rating and the scoring, contributing to over- or under-interpretation of the outcomes and prompting a misleading assessment (Reaven et al., 2008). Another limitation that can cause ADOS’ unreliable outcomes and affect the truthfulness of responses is the social desirability bias (Paulhus, 1991). Social desirability is a response bias in which individuals attempt to answer to tasks or questions in a manner that will be viewed as favorable by others (Edwards, 1957). First, part of the ASD assessment consists of reporting child information by family caregivers, who can interpret differently specific behaviors according to their personal perspective and experience (Möricke et al., 2016). Second, in ASD assessment, children may have been taught to act according to specific settings (e.g., laboratory settings) (Francis, 2005), and it might be that whether the same situation happened in the real world, examiners would obtain different responses (Gillberg and Rasmussen, 1994). Finally, although diagnostic structured interviews are considered as the gold standard in ASD assessment (Goldstein et al., 2009), they usually take place in the laboratory rather than in ecologically valid settings. Ecologically valid settings are environments and situations similar to real ones, able to elicit everyday experiences and behaviors related to daily functioning (Franzen and Wilhelm, 1996; Chaytor et al., 2006). The more the assessment measure is valid from an ecological point of view, the more that the results can be generalized to the real world (Brunswik, 1955; Chaytor et al., 2006). Indeed recent studies showed that traditional assessment results did not reflect performance in real-life situations and *vice versa* (Parsons S., 2016).

**FIGURE 1:**
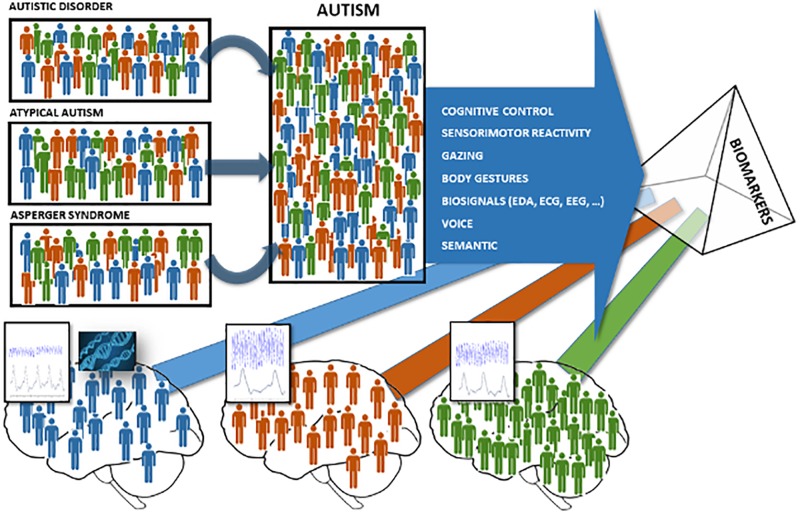
Biomarker models to classify neurodevelopment disorder populations. To the left and center: the three colors (red, blue, and green) represent the possible fault using the qualitative traditional assessment methods to classify the appropriate neurodevelopment disorder well according to the DSM-V. To the right and bottom: the three colors (red, blue, and green) represent the possibility to use biomarkers to quantify and classify neurodevelopment disorder populations with accuracy.

According to these limitations, the existing ASD diagnosis criteria (DSM, ICD, ADOS, and ADIR) do not consider quantitative variations in symptom severity in each person’s measurements and do not take into account the biological bases of the disorder. Recently, there has been a growing need for more reliable and valid diagnostic measures, such as biomarkers, to distinguish typical and atypical functioning and to reliably track the progression of the illness, thus helping to diagnose ASD (Figure 1). In order to generate valid quantitative models between explicit symptoms and implicit biomarkers, the emerging field of Computational Psychiatry (CP) is seeking, first, to mathematically model brain responses to the problems it faces and, second, to study how the “abnormal” experiences, emotions, and behaviors that are commonly used to describe disorders contribute to normal function and neural processes (Montague et al., 2012; Friston et al., 2014; Wang and Krystal, 2014; Redish and Gordon, 2016).

### Implicit Processes as Pillars for ASD Biomarkers

Currently, the EU AIMS Longitudinal European Autism Project is one of the largest multicenter, multidisciplinary studies to identify the stratification biomarkers for ASD and the biomarkers that may serve as surrogate ends (Murphy and Spooren, 2012). However, all participants are comprehensively characterized in terms of their brain structure and function [assessed using structural magnetic resonance imaging (sMRI), functional MRI (fMRI), and electroencephalogram (EEG)], biochemical biomarkers, prenatal environmental risk factors, and genomics. Nonetheless, when experiencing social situations, it is equally important to study the related behavioral outputs. Up to now, most of the information contained in the behavioral inputs do not seem to have been noticed. Studying social situations on how people process, store, and apply data about other people and social circumstances can provide us with objective information about the ASD evaluation.

Recent progress in social cognitive neuroscience (SCN), a field of study including biological processes and cognition-based aspects (Lieberman, 2010), is confuting the majority of social cognition models that suggest that humans can analyze and correctly verbalize their beliefs, feelings, and behaviors (Nosek et al., 2011), showing that our social interactions are mostly governed by unconscious processes that happen without conscious awareness or control (Forscher et al., 2019). To study the unconscious processes, several implicit measures, including brain images, behavior, and psychophysiological tracking, have been developed as alternative research methods to explicit measures since they are able to capture implied brain processes (Ledoux et al., 2016).

In the ASD population, implicit measures can contribute, along with traditional techniques, to obtain a more objective assessment from a quantitative point of view (Alcañiz et al., 2019). Various techniques used are based on measurements linked to some implied system in effect. The adoption of implicit SCN metrics as biomarker input variables for ASD evaluation suggests a move toward a quantitative ASD diagnosis. Some previous studies proposed the use of brain activity (fMRI and EEG), physiological measures (heart variability—HR), and behavioral responses (eye tracking measures—ET—body movement recognition), with the goal of capturing the ASD patient’s behavioral structure while being subjected to a stimulus (Di Martino et al., 2014; Van Hecke et al., 2015; Chita-Tegmark, 2016; Wang et al., 2016; Großekathöfer et al., 2017). For example, brain activity studies showed that ASD patients using fMRI present general brain hyperactivity and alterations in the middle and the posterior insula and in the cingulate posterior cortex (Di Martino et al., 2014). EEG studies in ASD showed greater activity in the left hemisphere in social situations (Van Hecke et al., 2015). In ASD, the study of gaze activity measured by eye tracking tools was analyzed as behavioral tests, linking the gaze patterns to the existence of nuclear deficits. Many studies have succeeded in linking this implicit measure with the affectation core deficits, with the degree of social, emotional, and cognitive skill development. Even in circumstances of social participation, predictors of ASD were found based on ocular actions and facial processing (Chita-Tegmark, 2016).

### Electrodermal Activity in ASD

To date, electrodermal activity (EDA, Nikula, 1991), a marker of sympathetic nervous system arousal, is one of the main implicit measures examined in ASD (White et al., 2014; Fenning et al., 2017). Specifically, it is an implicit neurophysiological process related to electrical proprieties of the skin, based on variations in sweating, skin conductance, heart rate, and blood flow to muscles when individuals are facing either internal or external stimuli (Fagius and Wallin, 1980; Benedek and Kaernbach, 2010; Boucsein, 2012). Its analysis allows to discern, among others, the phasic component of the signal, with rapidly changing activity, referred to the subject’s responses to discrete stimuli (being an indicator of sympathetic activity), and the tonic component, with slowly changing activity, referred to the subject’s basal conductance level (Dawson et al., 2007).

Regarding sensory dysfunction in ASD, multiple studies have investigated its relationship with EDA, comparing baseline arousal and EDA reactions to sensory stimuli among ASD individuals, neurotypical development population, and other diagnostic groups (for reviews, see Rogers and Ozonoff, 2005; White et al., 2014; Lydon et al., 2016). The evidence from these studies are controversial: some research found no differences in EDA levels in response to sensory stimuli (e.g., Zahn et al., 1987; Rogers and Ozonoff, 2005; McCormick et al., 2014), whereas other studies were successful (van Engeland et al., 1991; Miller et al., 2001; Rogers and Ozonoff, 2005; Schoen et al., 2009).

Overall regarding auditive stimulation, the enhanced EDA levels in ASD individuals have been associated to both baseline arousal and reaction to stimulus presentation (Palkovitz and Wiesenfeld, 1980; Barry and James, 1988; Chang et al., 2012); nevertheless, there are also instances about no differences between ASD and typical populations (e.g., Stevens and Gruzelier, 1984; Allen et al., 2013). Moreover, same pattern of mixed results has been found for immediate EDA of ASD individuals in visual stimulations: regarding reactions to facial expressions, autistic people exhibited weakened EDA responses compared to typical adults and children (Hirstein et al., 2001; Hubert et al., 2009; Riby et al., 2012), whereas Ben Shalom et al. (2006) found no differences; likewise, several studies related increased EDA reactivity to direct eye gaze in children with ASD (Kylliainen and Hietanen, 2006; Joseph et al., 2008; Kylliainen et al., 2012), but, conversely, other investigations did not (Louwerse et al., 2013). Furthermore, regarding smell processing, the ASD children seemed to be more sensitive than the TD children (Schoen et al., 2009); thus, they can detect odors at shorter distances (Ashwin et al., 2014); on the other hand, they have difficulties in detecting odor threshold (Dudova et al., 2011).

Finally, the correlation between ASD traditional assessments and EDA measures has been studied, observing that higher levels of ASD symptoms, measured by ADOS, are related to greater variability in EDA (Fenning et al., 2017).

### Use of Virtual Reality in ASD

To date, the above-described implicit measuring methodologies can be divided into two groups: studying the actions of the subject in a real scenario or conducting experiments in laboratory settings. The main problem with actual real-life scenarios is that it is not easy to study human responses in real situations because the experimenter struggles to fully monitor the stimuli involved in the encounter. Conversely, participants face controlled conditions in laboratory settings that do not include certain variables present in real-life situations, resulting in the experiment’s low ecological validity.

Virtual reality (VR) emerges as a promising technology capable of overcoming the problems mentioned above. VR offers the opportunity to create different real situations, including social situations that produce body interactions in which the body, environment, and brain are closely related. VR can be described as a virtual 3D environment that can replicate real experiences where participants can interact as if they were in the real world. Different technical tools can create a sense of presence, enabling the subjects to view their behaviors as real (Slater, 2009). Experiencing a high sense of presence enables the participants in the virtual environment (VE) to communicate and behave as if they were thinking, acting, and communicating in their real life (Alcañiz et al., 2019). Therefore, actions, attitudes, and beliefs can be transferred from nature to virtuality and *vice versa* and can occur spontaneously and unconsciously, generating circumstances of high ecological validity and maintaining high experimental control in stimuli presentation and in gathering behavioral performance. Neuroscientists are increasingly using VR to replicate natural phenomena and social interactions, developing immersive and multimodal sensory stimuli that provide advantages over real-life and traditional testing methodologies on the controlled stimuli and accuracy in data gathering (Bohil et al., 2011) and allowing also the integration of behavioral measures.

The use of VR in ASD research has been postulated as one of the methods with great potential in the treatment of the main symptomatological nucleus (Wing et al., 2011; Parsons T. D., 2016; Golestan et al., 2018). Such advantages have the theoretical basis established by Blascovich et al. (2002), who argued that interactive VEs would be able to change interaction and evaluation by offering the opportunity to study human behavior in normal, controlled, and replicable environments to produce an individual response close to that obtained in a real context.

One of the aims that are replicated for ASD and VR users throughout the research is to improve their ability to work in everyday life. Research that has built VE to learn different skills in children with ASD are not difficult to find: cognitive learning (Kandalaft et al., 2013), interaction (Bernardini et al., 2014), and emotional training (Bekele et al., 2014).

Nevertheless, there is a lack of research applied to the diagnosis in the field of VR in which an objective assessment of ASD is conducted through individualized clinical tests (behavioral biomarkers), customizing the treatment to each patient’s profile.

To our knowledge, no one has investigated whether multimodal VR settings and EDA reactions might contribute to predicting ASD population versus TD children. Starting from these premises, we performed two studies (the first exploratory and the second confirmatory) to discriminate and predict sensory processing in the ASD population versus in a TD population through the combined use of implicit measure (EDA) and different sensory stimuli, involving two different VE and tasks.

To this extent, the first experiment aimed to analyze the influence of three factors in predicting ASD: (1) the VE contents, one VE including a relaxing environment and another one including an arousal environment; (2) the task, one related to the subject’s greeting responses in the relaxing environment and others related to the subject’s imitation in the arousal environment; and (3) the stimuli conditions (SC), including visual (V), visual and auditive (VA), and visual, auditive, and olfactive stimuli (VAO). Specifically, in the first environment, the participants have been projected into a forest wherein the visual stimulus was a girl avatar appearing, the auditive stimulus was the sound of the rain, and the olfactive stimulus was the odor of fresh-cut grass. In this relaxing environment, the subjects were asked to complete tasks related to responding to the greetings of the avatars. In the second environment, the participants were introduced in a city street intersection in which the visual stimulus was the presence of two avatars (a girl and a boy), the auditive stimulus was a song that avatars danced to, and the olfactive stimulus was the smell of butter related to avatars that bit a muffin. In this arousal environment, the subjects were asked to complete a task related to the imitation of the actions of the avatars. In both environments and experiments, the EDA responses were recorded and introduced in a supervised machine learning classifier in order to recognize ASD.

Starting from these premises and aims, the first hypothesis in experiment 1 was that the ASD recognition is higher in the forest since the response to a greeting is one of the confirmatory symptoms in the ASD. The second hypothesis was that, by including more sensory modalities, the ASD recognition using EDA would present a better performance. After that, we performed a second experiment in order to develop a supervised learning model using the outputs of the first experiment. We increased the number of subjects used to calibrate the model and we tested it in a set of subjects not used before, simulating a real-world application.

## Materials and Methods

### Experiment 1

#### Participants

The study included 52 children between the ages of 4 and 7 years. In detail, 23 TD children (age = 4.87 ± 0.92; male = 13, female = 10) and 29 children with a previous diagnosis of ASD (age = 5.20 ± 1.34; male = 26, female = 3) participated in experiment 1. The ASD group sample was recruited from the Development Neurocognitive Centre, Red Cenit, Valencia, Spain. The ASD and the TD participants presented individual assessment reports that included the results of their ADOS-2 test. A sample management company recruited the TD group through targeted mailings to families. Before participating in the study, the family caregivers received written information about the study and they were required to give written consent for inclusion in the investigation. The study obtained ethical approval from the Ethical Committee of the Polytechnic University of Valencia. Furthermore, all procedures performed in the study involving human participants were in accordance with the ethical standards of the institutional and/or national research committee and with the 1964 Helsinki Declaration and its later amendments or comparable ethical standards.

#### Psychological Assessment

The following scales and tasks have been administered to the participants and their family caregivers:

• Autism Diagnostic Interview-Revised (ADI-R): The ADI-R (Lord et al., 1994) is a clinical semi-structured interview used to detect ASD and answered by family caregivers. The questions are linked to ICD-10 and DSM-IV criteria for autism and yield separate scores in three domains—communication, social interaction, and restricted, repetitive, and stereotyped behaviors. The answers are scored on a 0–3-point scale, in which 0 indicates the absence of the behavior and 3 indicates the clear manifestation of the determined behavior. ADI-R presents high psychometric properties and the test–retest reliability ranged from 0.93 to 0.97.
• Autism Diagnostic Observation Schedule (ADOS-2): The ADOS-2 (Lord et al., 1999) includes structured and semi-structured tasks to assess children’s development in several areas, such as communication, use of imagination, social interaction and play, and restrictive and repetitive behaviors. The measure uses five modules, tailored to the age and communication development of the participants. Concretely, module T is for young children who are between 12 and 30 months old and do not use phrase language consistently, module 1, for children who are 31 months or older and who do not use phrase language consistently, module 2 for children of any age who use phrase language but who do not have verbal fluency, module 3 for children with fluent language and young adolescents (under 16), and finally module 4 for adults and adolescents (16 years and older) with fluent language. From the observation of these behaviors, the items are scored between 0 (no evidence of abnormality related to autism) and 3 (definitive evidence), and from the sum of scores, two specific indices (social affectation and restricted and repetitive behavior) and the ASD global total index are obtained. The ADOS-2 presents excellent psychometric properties: the test–retest reliability is 0.87 for the social affectation index, 0.64 for the repetitive behavior index, and 0.88 for the total global index. In the study, the assessment was performed using module 1, corresponding to children from 31 months of age who do not use phrase language consistently.

#### The Virtual Environments

**FIGURE 2:**
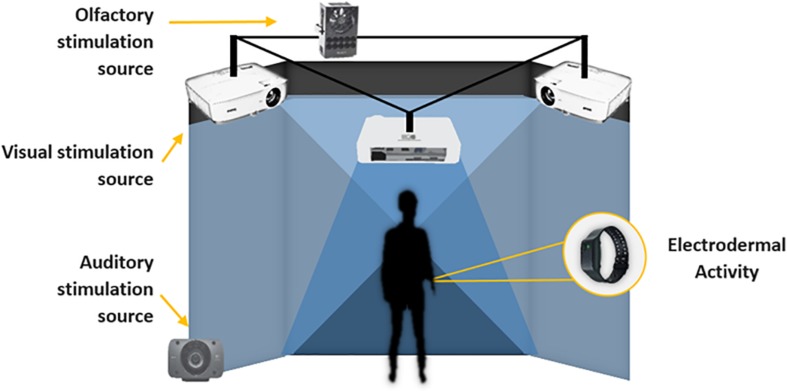
Experimental setting.

The 3D models were developed in the Institute for Research and Innovation in Bioengineering (i3B) at the Polytechnic University of Valencia. The environment was developed and projected inside a three-surface Cave Assisted Virtual Environment (CAVE^TM^) with dimensions of 4 m × 4 m × 3 m. It was equipped with three ceiling ultra-short lens projectors, which can project a 100° image from just 55 cm. The sound system used was the Logitech Speaker System Z906 500W 5.1 THX Digital (Figure 2).

**FIGURE 3:**
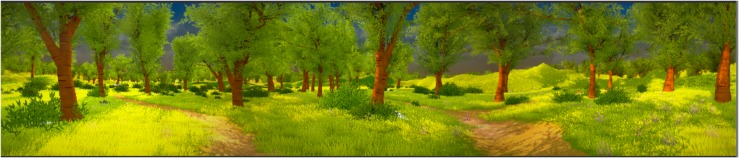
Virtual forest.

**FIGURE 4:**
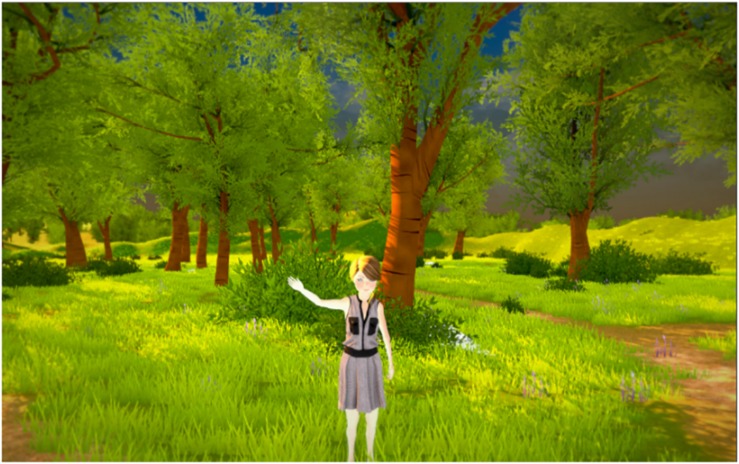
Girl’s avatar saying hello.

**FIGURE 5:**
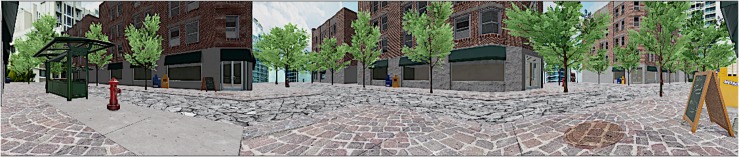
Virtual city street intersection.

**FIGURE 6:**
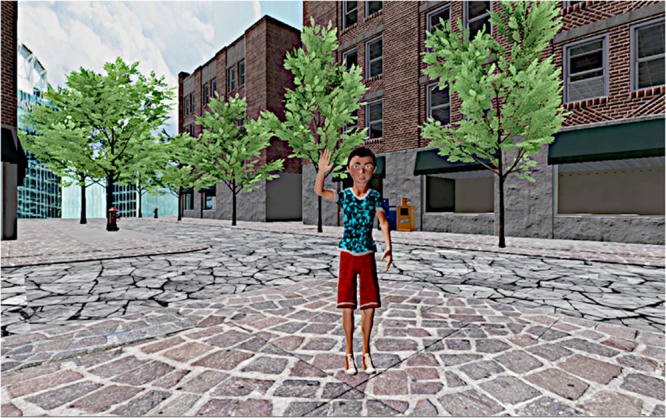
Boy’s avatar saying hello.

Two VEs were developed:

1. A virtual forest, including three controlled stimuli conditions: visual, visual–auditive, and visual–auditive–olfactive (Figure 3). The visual stimuli consisted of a girl’s avatar appearing from the left side of the forest and walking to the central virtual scene, where she stopped and waved her hand three times to say hello to the child, and then leaving the virtual scene, walking to the right side of the forest (Figure 4). The auditive stimuli consisted of adding to the virtual forest a storm and rain sound. Finally, the olfactive stimuli consisted of an odor of fresh-cut grass.

2. The other VE involved a simulated city street intersection (Figure 5) and was divided into three experimental stimuli conditions: visual, visual–auditive, and visual–auditive–olfactive. First, in the V stimuli condition, a boy’s avatar appeared from the left side of the surface CAVE^TM^, walking to the center of the virtual scene, where he stopped and waved his hand three times to say hello to the child, and then leaving the virtual scene, walking out of the street intersection (Figure 6). Successively, a girl’s avatar appeared in the central of the surface CAVE^TM^, walking to the right of the virtual scene, where she stopped and repeated the three waves with her hand to say hello to the child, and then leaving the virtual scene, walking to the right side of the street intersection. This sequence was repeated three times. In the second VA stimuli condition, the same avatars appeared in the same order from the same directions, but instead of waving the hand to say hello, they danced over a piece of music for 10 s for three times. In the VAO stimuli condition, the same avatars appeared in the same order and from the same directions, but they bit a buttered muffin, accompanied by the same song of the previous condition and an artificial butter smell that was released during the VR experience.

To avoid transfer effects over VR experiences, the VE presentation (forest and city street intersection) was counterbalanced across participants and a 1-week rest was left between the two VR experiences. Despite counterbalancing practice is also recommended for stimuli conditions of VEs, to reduce the possibility to provoke sensory sensitiveness overload in ASD children, the same stimuli presentation order was maintained (V, VA, and VAO) for the entire sample in both VR experiences. Indeed sensory sensitiveness in ASD can suddenly emerge in different situations that require the processing capacity of sensory integration from several channels (Bogdashina, 2016); such concurrent sensory decoding of stimuli might yield ASD children distress and uncomfortable states that could affect the quality of performance and assessment in VEs.

#### Physiological Assessment and Data Processing

Electrodermal activity signal was recorded using an Empatica E4 wristband.^1^ Its reliability has been found to be comparable to clinical devices in appropriate circumstances (McCarthy et al., 2016). Raw signal (recorded at 4 Hz and 0.001–100 μS) was pre-processed and analyzed using Ledalab^2^ (v.3.4.8) via Matlab^3^ (v.2016a). Pre-processing consisted of two successive phases: (1) Butterworth low-pass signal filtering at 2.5 Hz (Valenza and Scilingo, 2013) and (2) visual diagnosis of artifacts and their corrections. Due to the records characteristics and the analysis chosen, it was not considered necessary to apply signal-smoothing techniques. The analysis was tackled through the continuous decomposition analysis (CDA) method. It is based on the deconvolution of the skin conductance signal by the general response shape, prior to the data decomposition in the tonic and phasic components. As mentioned above, the tonic component generates slow changes in the conductance signal (magnitude of minutes), being considered the basal activity, and the phasic component generates rapid changes in the conductance signal (magnitude of seconds), being considered the response of the subjects to discrete stimuli. CDA has been proven to be an appropriate method for the analysis of short intervals between stimuli, especially in situations that can generate a high phasic activity (Benedek and Kaernbach, 2010). In order to reduce inter-subject differences, all values were standardized according to Venables and Christie (1980). This process was applied to the subject’s whole experience record. Finally, the set of metric extracted to characterize each stimuli condition includes the mean of tonic (BL tonic) and phasic (BL phasic) component of the baseline performed previously to the stimuli condition, the mean of tonic and phasic component of the responses to the stimuli condition, and the ratio between the tonic and the phasic component of the responses to the stimuli condition.

#### The Olfactive System

For the olfactive stimulus, we used the Olorama^4^ Technology^TM^ wireless freshener. It features 12 scents arranged in 12 pre-charged channels, which can be selected and triggered by means of a UDP packet. The device encompasses a programmable fan time system that dissipates the scent. Both the intensity of the chosen scent (amount of time the scent valve was open) and the amount of fan time were programmed. The scent valve was opened all the time during the last stimuli condition (VAO).

#### Experimental Procedure

**FIGURE 7:**
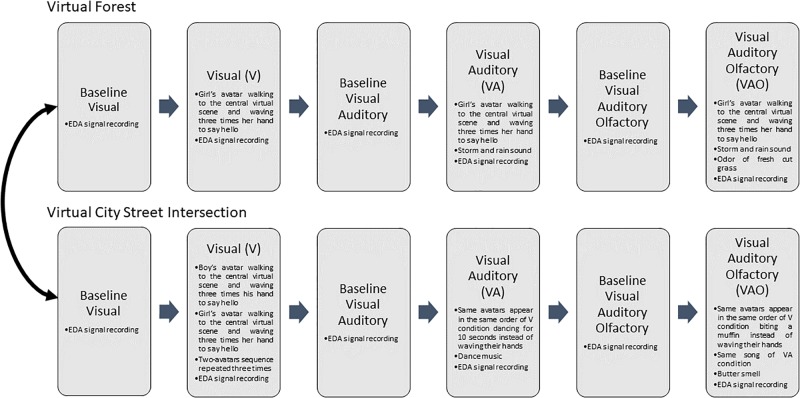
Experiment 1 procedure.

First, the family caregivers of the participants were informed about the general objectives of the research, the physiological measure and its device localization, and the VR system. Second, the Empatica E4 device was shown and placed on the participant’s non-dominant hand before the virtual session. Subsequently, the child was accompanied in the CAVE by the researcher and by his or her family caregiver, according to the child’s needs, and was placed in the middle of the virtual room, standing in front of the central surface at a distance of 1.5 m. Before each stimuli condition, 2 min of EDA baseline was recorded in rest and relaxing state, and then the VE experiences started (Figure 7). The total duration of the forest VE experience was 8 min and 15 s, and each stimuli condition lasted 45 s. The total duration of the city VE was 14 min, and each stimuli condition lasted for 2 min and 40 s. The participants were balanced between the two VEs, leaving a 1-week rest between the two experimental sessions.

During the three VR stimuli conditions in both virtual experiences, the EDA signals were recorded. The researcher monitored the child state during the entire experiment, and care was taken to address any indisposition derived from the use of the devices.

### Experiment 2

#### Participants

The study added 40 children, between the ages of 4 and 7 years, to experiment 1. In detail, 23 TD children (age 4.86 ± 0.91; male = 13, female = 10) and 17 ASD children (age 5.13 ± 1.35; male = 14; female = 3) participated in experiment 2. The ASD group sample was recruited from the Development Neurocognitive Centre, Red Cenit, Valencia, Spain. The ASD and the TD participants presented an individual assessment report that included the results of their ADOS-2 test. A sample management company recruited the TD group through targeted mailings to families. Before participating in the study, the family caregivers received written information about the study, and they were required to give written consent for the inclusion in the investigation.

#### Psychological Assessment

Experiment 2 utilized the same scales and tests of experiment 1.

#### Physiological Assessment and Data Processing

The EDA signals were recorded using Empatica E4 wristband, as in experiment 1,^5^ and the physiological data processing and analyses were performed using the method described in the “Physiological Assessment and Data Processing” section of experiment 1.

#### The Olfactive System

The system used was the same as that implemented in experiment 1: the Olorama Technology^TM^^6^ wireless freshener.

#### Experimental Procedure

In experiment 2, the participants only experienced the forest VE as follows: first, as in experiment 1, the family caregivers of the participants were informed about the general objectives of the research, the physiological measure and its device localization, and about the VR system. Second, the Empatica E4 device was shown and placed on the participants’ arm of the non-dominant hand before the virtual session. Consequently, the child was accompanied in the CAVE by the researcher and by his or her family caregiver according to the child’s needs. The participant was placed in the middle of the virtual room, standing in front of the central surface at 1.5 m. Firstly, 2 min of EDA baseline was recorded in resting and relaxing state. Next, the three stimuli VR experience conditions were presented, recording a 2-min EDA baseline before each one.

### Statistical Analysis

In experiment 1 (*n* = 54), three participants were excluded from the analysis for lack of EDA data due to bad recording: two from the forest VE and one from the city VE. Consequently, the sample size included 52 children for forest VE analysis and 51 in the city VE. In this preliminary stage, we developed four models for each environment (forest VE and city VE) in order to explore the importance of the scenario and each stimuli condition (SC). The first model included all the SC, the second only the visual stimuli, the third only the VA stimuli, and the fourth only the visual, auditive, and olfactive SC. Moreover, we developed two extra models to analyze if they can achieve a performance better than chance. To this extent, we computed a permutation-based test, i.e., we developed two models with city/forest VE input data (all stimuli) with a random output class assignment. The development of the models (parameter tuning and feature selection) used cross-validation with all the samples of the experiment. To compare model performance, we used the output of the classification algorithm without bipolarization, i.e., the probability between 0 and 1 that the model as their true class classified a subject. Due to the Gaussianity of the data (*p* > 0.05 from the Shapiro–Wilk test with null hypothesis of having a Gaussian sample), we performed a statistical model comparison using the probabilities of the models by applying a one-way ANOVA with Tukey–Kramer correction.

In experiment 2 (*n* = 92): in order to calibrate and test the final model, we used the case of the forest VE and all the SC, increasing the participants to boost the final model and test it. We split the dataset into a training set (*n* = 72) and a test set (*n* = 20). The test set was sliced randomly using the new subjects, but keeping a balanced 50% of each class. The development of the model (parameter tuning and feature selection) used cross-validation with the training set and afterward was applied in the test set that has not been previously used.

To develop the models, we used support vector machine (SVM)-based pattern recognition (Schölkopf et al., 2000) with a leave-one-subject-out (LOSO) cross-validation procedure. For the LOSO scheme, the training set was normalized by subtracting the median value and dividing by the median absolute deviation over each dimension. In each iteration, the validation set consisted of one specific subject and he/she was normalized using the median and deviation of the training set. In particular, we used an optimized C-SVM using a sigmoid kernel function, changing the parameters of cost and gamma using a vector with 15 parameters logarithmically spaced between 0.1 and 1,000. Moreover, we performed a feature selection strategy to explore the relative importance of each feature. A support vector machine recursive feature elimination (SVM-RFE) procedure, in a wrapper approach, was included (RFE was performed on the training set of each fold and we computed the median rank for each feature over all folds). We specifically chose a recently developed, non-linear SVM-RFE, which includes a correlation bias reduction strategy in the feature elimination procedure (Yan and Zhang, 2015). The model was optimized to achieve best Cohen’s kappa. The algorithms were implemented using Matlab© R2016a and LIBSVM toolbox (Chang and Lin, 2011).

## Results

### Experiment 1: Model Comparisons

**FIGURE 8:**
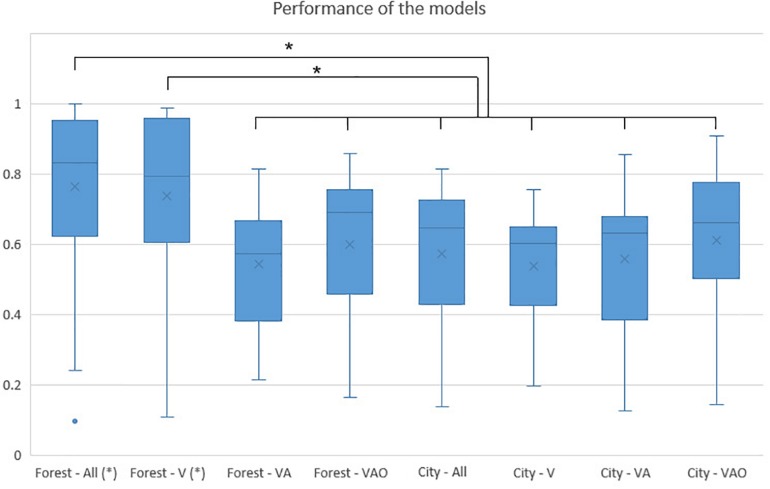
Comparison of performance model. Bars represent the means of the probability (between 0 and 1) that a subject was classified by the model as their true class; vertical lines represent the standard deviation of the means; asterisk indicates significant differences with *p* < 0.05.

Table 1 shows the performance of the eight models performed, considering both VEs and SC. It includes the accuracy of each model, the confusion matrix, and the features included derived to the automatic feature selection procedure. In addition, Figure 8 shows a comparison of the performance of each model, considering the probability that the model as their true class classified a subject and the significant differences between models were derived from one-way ANOVA using a Tukey–Kramer correction. We included in the ANOVA 2 permutated models to test if the accuracy is significantly better than chance, where the accuracy is 67.30% for the forest VE and 68.62% for the city VE. The result of the one-way ANOVA shows that there are differences between models (*p* < 0.0001).

**TABLE 1:** Overview of the performance of the models including total accuracy, Cohen’s kappa, permutation test (*shows significant differences), true positive, and true negative.

						Features included in the model														
				Confusion matrix		Visual stimuli condition (V)					Visual and auditive stimuli condition (VA)					Visual, auditive, and olfactive stimuli condition (VAO)				
							
Study	Acc (%)	Kappa	Permutation test	TPR (%)	TNR (%)	BL tonic	BL phasic	Tonic	Phasic	Ratio	BL tonic	BL phasic	Tonic	Phasic	Ratio	BL tonic	BL phasic	Tonic	Phasic	Ratio
Forest—all (*n* = 52)	90.38	0.806	*	89.66	91.30	X	X		X	X					X	X		X		
Forest—V (*n* = 52)	84.62	0.691	*	82.76	86.96	X	X				–	–	–	–	–	–	–	–	–	–
Forest—VA (*n* = 52)	71.15	0.418	–	72.41	69.57	–	–	–	–	–		X		X	X	–	–	–	–	–
Forest—VAO (*n* = 52)	75.00	0.496	–	75.86	73.91	–	–	–	–	–	–	–	–	–	–	X				
City—all (*n* = 51)	70.59	0.397	–	75.86	63.64									X		X		X	X	X
City—V (*n* = 51)	68.63	0.323	–	89.66	40.91					X	–	–	–	–	–	–	–	–	–	–
City—VA (*n* = 51)	72.55	0.415	–	89.66	50.00	–	–	–	–	–				X		–	–	–	–	–
City—VAO (*n* = 51)	76.47	0.520	–	79.31	72.73	–	–	–	–	–	–	–	–	–	–		X	X	X	X

*Features included in the models have been marked with an (x), features proposed but not included (i.e., not selected by the automatic feature selection procedure) have been marked with a blank space (), and features excluded *a priori* of the model have been marked with a dash (–). Acc, accuracy; TPR, true positive rate; TNR, true negative rate; BL, baseline.*

The highest accuracy (90.3%, kappa = 0.80) was achieved by experiment 1 including all SC and it presented higher performance than the rest of the models (forest—VA *p* = 0.000, forest—VAO *p* = 0.000, city—all *p* = 0.000, city—V *p* = 0.000, city—VA *p* = 0.000, city—VAO *p* = 0.002), except with the forest—V SC where no statistical significance was found. The model included four features of the V SC (baseline tonic, baseline phasic, phasic, and ratio), one feature of the VA SC (ratio), and two features of VAO SC (baseline tonic and tonic). The second highest accuracy (84.6%, kappa = 0.69) was achieved by the forest including V SC, and it presented a higher performance than the rest of the models with lower accuracy (forest—VA *p* = 0.000, forest—VAO *p* = 0.008, city—all *p* = 0.001, city—V *p* = 0.000, city—VA *p* = 0.000, city—VAO *p* = 0.029). The model only included two features (baseline tonic and baseline phasic). Both models have a strongly balanced confusion matrix. These two models presented a performance statistically different than chance (forest—all *p* = 0.000 and forest—V *p* < 0.0001).

The rest of the models presented accuracy between 68 and 76% and did not present statistically significant differences in terms of performance between them and the permutated models. The model, including the VA stimuli condition of the forest, showed an accuracy of 71.15% (kappa = 0.41) and included the three features (phasic baseline, phasic, and ratio). The model, including VAO SC of the forest, achieved a balanced accuracy of 75.00% (kappa = 0.49), including only the tonic responses in the baseline. Regarding the city VE, the model included all stimuli conditions, achieved 70.5% (kappa = 0.39) using one feature of VA SC (phasic) and four features of VAO SC (baseline tonic, tonic, and phasic and ratio). The models, including the V and the VA SC, achieved 68.63% (kappa = 0.32) and 72.55% (kappa = 0.41) of accuracy respectively, but with a very bad balance in terms of false positives. The model, including the VAO SC, achieved a balanced 76.47% (kappa = 0.52) of accuracy including four features (baseline phasic, tonic, phasic, and ratio).

### Experiment 2: Development of the Final Model

**FIGURE 9:**
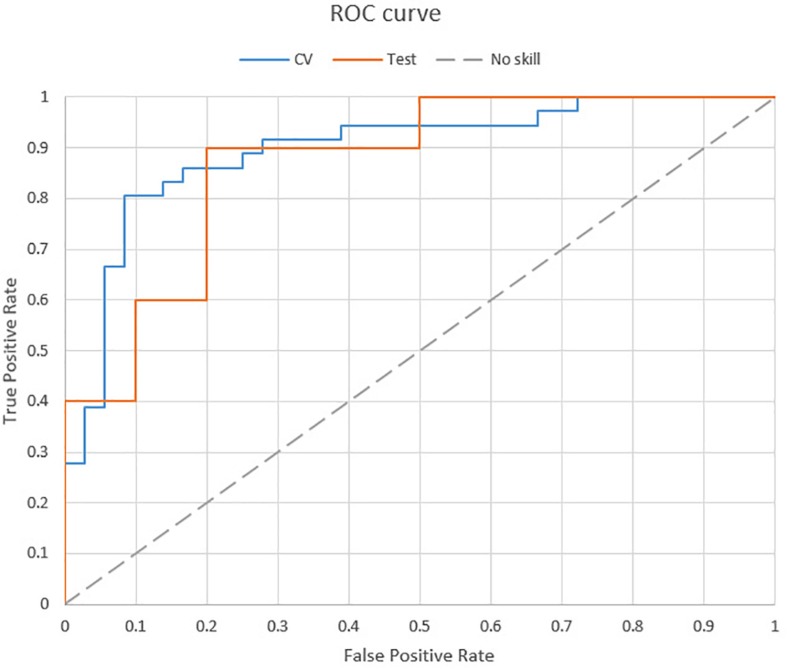
ROC curve of the final model.

Table 2 shows the performance of the final model derived from the forest VE after the increment of the subjects. The validation set (*n* = 72) shows a balanced accuracy of 83.33% (kappa = 0.668). The test set (*n* = 20) achieved 85% of accuracy (kappa = 0.700), recognizing 80% of subjects with sensory dysfunction. The model included one feature of the V SC (phasic), three features of the VA SC (baseline tonic, baseline phasic, and phasic), and one feature of the VAO SC (baseline tonic). In addition, Figure 9 shows the ROC curve of the performance of the final model, achieving an area under a curve (AUC) of 0.897 in the validation and 0.870 in the test.

**TABLE 2:** Overview of the performance of the final model including accuracy, AUC, Cohen’s kappa, true positives, true negatives, and features included in the models (marked with an X), considering validation and test set.

						Features included in the model														
				Confusion matrix		Visual phase (V)					Visual and auditive phase (VA)					Visual, auditive and olfactive phase (VAO)				
							
Study	Acc (%)	Kappa	AUC	TPR (%)	TNR (%)	BL tonic	BL phasic	Tonic	Phasic	Ratio	BL tonic	BL phasic	Tonic	Phasic	Ratio	BL tonic	BL phasic	Tonic	Phasic	Ratio
Forest—validation set (*n* = 72)	83.33	0.668	0.897	86.11	80.55				X		x	x		x			x			
Forest—test set (*n* = 20)	85.00	0.700	0.870	80.00	90.00															

*BL represents the responses recorded in the baseline.*

## Discussion

The main aim of this study was to discriminate and predict sensory processing, recognizing ASD population versus TD population through the combined use of implicit measure (EDA) and different sensory stimuli in VR. Specifically, two experiments have been run, testing two different VEs, presenting three sensory stimuli conditions each—visual, visual and auditive, and visual, auditive, and olfactive stimuli—and examining EDA changes before and during the presentation of the virtual and the sensory stimuli. The focus has been on sensory processing because there are evidences that it is relatively impaired in the ASD population (Leekam et al., 2007; Tomchek and Dunn, 2007; Baron-Cohen et al., 2009).

The results can be discussed on four levels: (1) the influence of scenarios and stimuli conditions, (2) the role of EDA and the features used, (3) the performance of ASD recognition, and (4) the limitations and further studies.

### The Influence of Scenarios and Stimuli Conditions

Regarding scenarios, the model developed using the forest VE presented a higher accuracy (forest VE—all, 90.3%) than the model developed using the city VE (city VE—all, 70.59%). Since we used the same set of subjects, the results are not influenced by the individuals’ bias. Therefore, a model comparison validated the hypothesis that ASD recognition was higher in the forest VE (forest VE—all vs. city VE—all, *p* = 0.000). Moreover, the permutated test shows that forest—all and forest—V are the models that statistically offer a performance better than chance. This outcome could be due to task characteristics since the response to a greeting is one of the confirmatory symptoms in ASD. In addition, several previous studies showed the influence of nature scenes in reducing arousal (Liszio et al., 2018; White et al., 2018). Therefore, the forest VE can be assumed as a more relaxed environment than the city VE. Since EDA is highly affected by the arousal (Picard et al., 2016), the results suggested that a natural and relaxed environment as a forest VE could be a better scenario to detect changes in the ASD population due to sensory processing dysfunctions. In addition to the increase of arousal derived from city VE, the avatars imitation task provoked a physical activity in the subject that could affect arousal, decreasing the recognition performance of models, due to an arousal saturating effect; hence, the results supported the use of a low-arousal natural environment and non-physical activities to increase the performance recognition of models using EDA.

Regarding stimuli conditions, the model developed in the forest VE with all the stimuli conditions achieved 5.78% of accuracy more than the forest with only visual stimulation, but it did not show statistical differences. Both models presented higher accuracy and performance than the rest of the models, including the permutated one. However, the model developed in the city VE used only one feature of V SC, three features of VA SC, and four features of VAO SC. Therefore, even though the exploratory analysis performed in forest VE suggested that VA and VAO did not play an important role in the ASD recognition in comparison with V, the feature selection for the final model showed high reliance on the multimodal sensory condition since four out of five of the features selected were from VA and VAO stimuli. The hypothesis that increasing sensory modalities would have contributed to better ASD recognition through EDA is partially confirmed by the final model.

### The Role of EDA and the Features Used

To our knowledge, we proposed the first supervised ML model using EDA for ASD population [see Hyde et al. (2019) for a review of ASD models recognition]. Our results were in accordance with previous research that showed that ASD is associated with the autonomic nervous system and can be measured using EDA (Miller et al., 2001; Rogers and Ozonoff, 2005; Schoen et al., 2009; Bujnakova et al., 2016). However, other researches did not find differences in EDA levels in response to sensory stimuli in the ASD population (e.g., Zahn et al., 1987; Rogers and Ozonoff, 2005; McCormick et al., 2014). The level of recognition of the presented models represents a new step in the use of the autonomic nervous system as a biomarker for ASD recognition. In addition, the CDA analysis showed a valid signal processing method to extract valuable features from EDA. The phasic responses of the subjects in the two SCs (V and VA) are included in the feature selection of the final model and in many of the exploratory model comparison. A baseline is also a very important part of the stimuli since the baseline responses of VA (tonic and phasic) and VAO (phasic) SCs were included in the final model. Moreover, the baseline responses were also included in forest VE—all and forest VE—V models of forest VE. In this regard, it should be noted that variations in the phasic and tonic components, related to changes in emotional arousal, have been reported by studies carried out in different experimental paradigms (Kreibig, 2010). The relevant role of the baseline was in accordance with previous research that suggested that the participants are likely to be hyper- or hypo-responders independent of any effects of stimuli (Braithwaite et al., 2013). Moreover, the role of baselines could be especially important in research on sensory processing disorders as in ASD.

### The Performance of ASD Recognition

Regarding the final model on ASD recognition, the validation set using 72 subjects achieved 83.33% of accuracy (kappa: 0.668, AUC: 0.897), including 86.11% of true positives. Moreover, we tested the model in a set of 20 subjects (10 ASD and 10 controls) recruited in a second phase, and the model achieved 85% accuracy (kappa: 0.700, AUC: 0.870). The results presented perform a new step in ASD recognition since, to our knowledge, we presented the first ASD-supervised ML recognition model using EDA and multimodal VR. Moreover, the methodology presented some advantages in contrast to previous research. Li et al. (2017) presented an analysis using kinematics recognizing ASD in adults achieving 86.7% accuracy (*n* = 30). Liu et al. (2016) developed a model using eye tracking to recognize ASD children based on face processing, achieving 88.51% of accuracy (*n* = 58). Nakai et al. (2017) presented an ASD model recognition in children using voice analysis, achieving 76% of accuracy (*n* = 30 ASD, *n* = 51 TD). All of them validated their models using cross-validation procedures and used ecological biomarkers to recognize ASD. Contrarily, the presented model achieved the same (or more) level of accuracy, but using a broader sample size and, moreover, applying the model to a new test set that was not used before, simulating a real application. It supposes a new step forward in order to develop scalable clinical applications of ASD recognition models. On the other hand, previous research by Chen et al. (2015) showed a very large study (*n* = 252) with a very high accuracy (91%) using fMRI. In contrast to this approach, we proposed an ecological environment and instrumentation using VR and EDA wristband sensor. This ecological approach is particularly important in the field of ASD and can offer cheaper and quicker clinical diagnostic models in the future.

### Limitations and Future Studies

Although this study did a step forward in the field of ASD sensory processing assessment, it presented some limitations regarding sample characteristics, specific ASD symptoms and their related measures, and VEs.

First, the participants were from 4 to 7 years old and selected ASD children received, according to their symptomatology and age, a previous ASD diagnosis through the module 1 of the ADOS-2 questionnaire that is addressed to infants older than 31 months of age but who do not use phrase language consistently. Nonetheless, it has been decided to test only participants who pertained to this class and characteristics to control and ensure results, but these narrow criteria limit the generalization of findings.

Second, the present study mostly focused on ASD sensory processing although it is not a core ASD symptom for diagnostic manuals, such as DSM-5 and ICD-10. Furthermore, regarding VEs, at the first time, children might experience them as astonishing and impressive (Novelty effect, Clark, 1983; Gravetter and Forzano, 2018); for this reason, in the first part of each study, there might be a common effect on EDA metrics, especially in forest-V and city-V conditions. However, this artificial activation arousal, that is due to the sense of being physically present in a VE despite the certainty of not being physically there, decreases as the familiarity with the virtual world and device increases. Third, the sample size was restricted and not matched on socio-demographics, limiting the generalization of the model outcomes. In accordance with limitations, future works are needed in order to develop an objective method for the assessment of sensory processing in the ASD population. Future studies must, first, include a broader sample size with further control and matching on sociodemographics. Socioeconomic status is recommended in order to avoid misleading model outcomes based on other metrics far from ASD presence or absence (Delobel-Ayoub et al., 2015). Second, ASD individuals should be diagnosed by the five modules of ADOS-2 questionnaire to test whether the results presented here may be generally replicated in all age range and linguistic ability clusters. Moreover, in conjunction with sensory measures, the inclusion of core symptom analyses in VR is suggested, for example, repetitive and stereotypical behaviors, and communication and social abilities. Biomarkers that could be relevant for this purpose are eye tracking, body movement analysis, and EEG (Loth et al., 2016); indeed eye tracking glasses and RGBD cameras for body movement analysis might be included in future studies on current VR experiences in order to enhance model strength and accuracy. Furthermore, to discern impaired sensory processing, the present study involved three VR conditions (visual, auditive, and olfactive) and it could be interesting to add a fourth condition about haptic processing since it enhances immersion in the VE, providing a more ecological and realistic experience (Slater and Wilbur, 1997). Finally, some adjustments of the virtual content might bring more sense of presence to the participants, such as the introduction, in both VA and VAO stimuli conditions, of auditive stimulation consistent with the avatar that is waving the hand to say hello.

## Conclusion

Sensory processing is a relevant ability in information processing, allowing adapting behavioral responses to the environment (Miller et al., 2007). ASD show hyper-sensitiveness (over-responsiveness) to VA stimuli and hypo-sensitiveness (under-responsiveness) to olfactive stimuli (Tomchek and Dunn, 2007; Baron-Cohen et al., 2009; Dudova et al., 2011; Ashwin et al., 2014; Tomchek et al., 2014). The hyper–hypo sensitiveness to sensory stimuli can generate an alteration in information processing, affecting cognitive and social responses in daily life situations. Traditional ASD assessment, based on semi-structured behavioral task observations on laboratory settings and structured interviews, does not take into account the dysfunctional sensory processing in real life. According to the results, current studies have shown that it is possible to obtain biomarkers for ASD classification using a CP paradigm based on implicit brain processes, measured through psychophysiological signals and the subjects’ behavior, while exposed to complex social conditions using VR interfaces. The ASD classification using biomarkers, along with traditional assessment, could enhance knowledge on the development of relevant specific treatments.

## Data Availability Statement

The datasets generated for this study are available on request to the corresponding author.

## Ethics Statement

The studies involving human participants were reviewed and approved by Polytechnic University of Valencia. Written informed consent to participate in this study was provided by the participants’ legal guardian/next of kin.

## Author Contributions

All authors have contributed to the manuscript as follows: MA and LA designed the study and supervised the whole study. EO and MS recruited and assessed the eligible subjects. JM-M conducted the odor and statistical analyses. IC, GT, MM, MS, JM-M, and JH-T wrote the original manuscript and MA and LA revised the manuscript. All authors assisted in the revision process, read, and approved the final manuscript.

## Conflict of Interest

The authors declare that the research was conducted in the absence of any commercial or financial relationships that could be construed as a potential conflict of interest.

## Footnotes

1 www.empatica.com

2 www.ledalab.de

3 www.mathworks.com

4 www.olorama.com

5 www.empatica.com

6 www.olorama.com
